# Neurodiversity in the teaching of the mental state examination: a pilot study of interactive mind-mapping seminars for the new ScotGem (Scottish graduate-entry medicine) students during the COVID-19 pandemic

**DOI:** 10.1192/bjo.2021.87

**Published:** 2021-06-18

**Authors:** Kathleen Breslin, Sara Mohsen, Praveen Kumar

**Affiliations:** 1 New Craigs Hospital, NHS Highland, University of Aberdeen; 2 New Craigs Hospital, NHS Highland

## Abstract

**Aims:**

Agility in educational delivery has been catalyzed in response to national restrictions mandated by the recent COVID-19 pandemic. Increased use of assistive technologies further aligns with the General Medical Council's aims that medical educators provide an 'accessible training experience'. The study examined medical students' receptiveness to different types of interactive teaching. Two undergraduate cohorts received teaching on the Mental State Examination, either socially-distanced delivered by traditional powerpoint or remotely by mind-mapping software on a tablet hand-held digital device. We required an effective program which would retain the popular interactive elements of Psychiatry teaching and promote inclusivity across students' diverse learning styles.

**Method:**

Two cohorts of Year 2 students from the Universities of Dundee and St Andrew's Scottish Graduate-Entry Medicine (scotGEM) course took part in an Introduction to Psychiatry seminar which involved a presentation of the Mental State Examination. One was conducted in a face-to-face setting via traditional PowerPoint. The second was conducted via remote-conferencing with mindmaps of key concepts drawn and screen-shared live to students as teaching progressed.

This was a qualitative study, with online links to questionnaires for 24 student participants across 5 domains. (1. The tutorial met my learning objectives, 2. The format was suitable for me, 3. The balance of theory and cases was suitable for me, 4. The tutorial was of appropriate length, 5. I was satisfied with the performance) Response options included: strongly disagree, disagree, neutral, agree, strongly agree. A section was also included with open-ended questions pooled for thematic analysis.

**Result:**

Response rate reached >60% with >80% respondents answering strongly agree across all domains. Thematic results demonstrated positive responses across both teaching sessions, with the interactive elements valued by students. Comments included: “great job was done with the delivery of the session considering it was online rather than in person”; “drawing element was fantastic”; “Good: interactivity of the session drawing and creativity element”.

**Conclusion:**

The Mental State Examination (MSE) via live-drawn mind-maps allows salient clinical information to be conceptualised in non-linear diagramatic format. This paediological approach can offer further access points across wide range of learning styles. This pilot study demonstrated such interactive components of Psychiatry teaching continue to be well received and can be effectively delivered remotely. Such sessions also serve to promote inclusivity, linking those who are geographically distant in addition to the visual learner and the neurodiverse. We aim to incorporate these dynamic teaching sessions into our online induction programs and disseminate Intelligent Tutorials to our remote and rural learners throughout Scotland.

